# PROMoting the use of studies within a trial (PROMETHEUS): Results and experiences from a large programme to evaluate the routine embedding of recruitment and retention strategies within randomised controlled trials routinely

**DOI:** 10.1177/26320843221147841

**Published:** 2022-12-22

**Authors:** Laura Doherty, Adwoa Parker, Catherine Arundel, Laura Clark, Elizabeth Coleman, Catherine Hewitt, David Beard, Peter Bower, Paul Brocklehurst, Cindy Cooper, Lucy Culliford, Declan Devane, Richard Emsley, Sandra Eldridge, Sandra Galvin, Katie Gillies, Alan Montgomery, Chris Sutton, Shaun Treweek, David Torgerson

**Affiliations:** 1 York Trials Unit, Department of Health Sciences, 8748 University of York, UK; 2 Nuffield Department of Orthopaedics, Rheumatology, and Musculoskeletal Science, NIHR Biomedical Research Unit, 6396 University of Oxford, UK; 3 National Institute for Health Research School for Primary Care Research, Centre for Primary Care and Health Services Research, 5292 University of Manchester, UK; 4 1506 Bangor University, Gwynedd, UK; 5 School of Health and Related Research, 7315 University of Sheffield, Sheffield, UK; 6 Bristol Trials Centre, Clinical Trials and Evaluation Unit, 1980 University of Bristol, UK; 7 School of Nursing and Midwifery, 8799 National University of Galway, Ireland; 8 Department of Biostatistics and Health Informatics, Institute of Psychiatry, Psychology and Neuroscience, 4616 King’s College London, UK; 9 Institute of Population Health Sciences, 4617 Queen Mary University of London, UK; 10 Health Services Research Unit, 1019 University of Aberdeen, Aberdeen, UK; 11 Nottingham Clinical Trials Unit, University Park Nottingham, 6123 University of Nottingham, UK; 12 School of Health Sciences, 5292 University of Manchester, UK

**Keywords:** Recruitment and retention < RESEARCH DESIGNS & METHODS, Studies within a trial, randomised controlled trial, RESEARCH DESIGNS & METHODS, methods & methodology < BASIC CONCEPTS OF RESEARCH

## Abstract

**Aim:**

PROMoting THE USE of Studies Within A Trial (PROMETHEUS) aimed to improve the evidence base for recruiting and retaining participants in Randomised Controlled Trials (RCTs) by pump-priming and facilitating the start of at least 25 Studies Within A Trial (SWATs) testing recruitment or retention interventions.

**Methods:**

Ten Clinical Trials Units (CTUs) and one Primary Care Research Centre formed a network to conduct randomised SWATs of recruitment and/or retention strategies. We identified promising recruitment and retention interventions from various sources, which were reviewed by patient and public (PPI) partners to generate an initial priority list of seven recruitment and eight retention interventions.

Host trial teams could apply for funding of up to £5000 and receive support from the PROMETHEUS team to design, implement, and report SWATs. We additionally tested the feasibility of undertaking coordinated SWATs across multiple host trials simultaneously.

**Results:**

PROMETHEUS funded 42 SWATs, embedded within 31 host trials, across 12 CTUs. The SWAT cost per SWAT was £3535. Of the 42 SWATs, 12 tested the same SWAT in multiple trials (*simultaneous SWAT design)* and eight tested a factorial SWAT design. PROMETHEUS will add 18% and 79% more SWATs to the Cochrane systematic review of recruitment strategies and the Cochrane review of retention strategies respectively.

**Conclusion:**

The PROMETHEUS programme substantially increased the evidence base for both recruitment and retention strategies within RCTs. Future research should adopt a systematic approach to identifying and targeting gaps in the evidence base and focus on translating SWAT evidence into recruitment and retention practice.

## Introduction

Randomised controlled trials (RCTs) are crucial for evidence-based healthcare. Despite substantial amounts of money being invested, many trials fail to recruit to time and budget, often with significantly higher attrition than anticipated. One review found that only 56% of RCTs achieve their planned sample size.^1^ The costs of poor recruitment can be huge^2^ and this constitutes significant research waste.^3,4^ Similarly, low participant retention reduces the power of a study and can cause the estimates of an intervention’s effect to be biased.^5^ A priority-setting exercise involving 85% of UK Clinical Trials Units (CTUs) placed recruitment and retention as the top two priorities for methodological research.^6^

Randomised controlled trials embedded within ‘host’ RCTs, otherwise referred to as ‘Studies Within a Trial’ (SWATs), are the most robust way of evaluating strategies for improving participant recruitment and retention in RCTs.^7^ Two Cochrane reviews identified 150 SWATs of strategies to increase recruitment and/or retention in RCTs^8,9^; however, effective, evidence-based strategies are rare. Where evaluations do exist, they tend to occur in the context of single RCTs, meaning their effects across different trial contexts are unclear^8^ and they are often poorly reported.^10^ The most recently published Cochrane review on retention interventions concluded that there was no high-certainty evidence for any of the evaluated strategies, as assessed by GRADE.^9^

Members of the PROMETHEUS team previously worked on the UK Medical Research Council (MRC) START project,^11,12^ a feasibility study that successfully developed the conceptual, methodological and logistical framework to improve recruitment through the embedding of 2 recruitment strategies in 12 host trials in primary care and developed reporting guidelines for embedded RCTs.^13–15^ In addition, since 2014, the Health Research Board – Trials Methodology Research Network (HRB-TMRN, Ireland), have supported and funded Irish researchers to conduct methodological studies to improve the efficient conduct of future RCTs including SWATS. The PROMETHEUS programme aimed to build on this work and make the embedding of SWATs within RCTs standard practice across multiple clinical trials units by pump-priming and facilitating the start of at least 25 SWATs within 30 months.

## Methods

### PROMETHEUS preparatory work

Before the initiation of the PROMETHEUS programme, we identified a network of eight CTUs, one primary care research centre in the UK, and the Health Research Board – Trials Methodology Research Network (HRB-TMRN) in the Republic of Ireland, who each committed to embedding either a recruitment and/or retention SWAT within at least two host trials.

Promising (meaning some evidence of benefit but with substantial uncertainty) recruitment and retention strategies were identified from a variety of sources by the York Trials Unit PROMETHEUS team and the programme co-applicants. These sources included Cochrane systematic reviews,^8,10,16^ the UK MRC SWAT Repository Store (SWAT store: www.qub.ac.uk/SWAT-SWAR), the priorities identified by CTUs of recruitment and retention strategies^17^ and the PRioRiTy list of top 10 unanswered questions on trial recruitment.^18^ A PPI panel was also convened to highlight the top priority strategies to be evaluated.

These specific strategies were prioritised if they met one or more of the following criteria:

(1). Previously reported peer-reviewed publications.
(2). Under current evaluation.
(3). Easy to implement within-host trials.
(4). Had the potential to significantly impact participant retention or recruitment (which are often the more challenging, expensive strategies to implement).
(5). Strategies identified by host trial teams as suitable for testing in a SWAT within their trial

These criteria were designed to capture a diverse range of recruitment and retention strategies whilst also identifying those which would contribute to the development of the existing evidence base and help derive definitive conclusions (subject to sufficient replication).

The prioritisation of these strategies was derived through group discussion and consensus. These priorities formed an initial strategy priority list of 7 recruitment and 8 retention strategies (Table 1), which was reassessed and rearranged accordingly throughout the programme, based on emerging SWAT evidence. The four criteria determined the ranking of specific strategies in Table 1.

**Table 1.** List of key recruitment and retention questions in priority order.

Recruitment strategies
1. What is the effect of adding a pen printed with the trial/university logo to the trial invitation on recruitment rates (SWAT 37)?
2. What is the impact of recruitment sites receiving an extra trial co-ordinator visit on recruitment rates (SWAT 27)?
3. What is the effectiveness of a brief participant information leaflet (PIL) versus standard length PIL on participant recruitment rates? (SWAT 137)
4. What is the impact of a training workshop for staff recruiting patients into trials on recruitment rates (SWAT 111)?
5. What is the effect of offering financial incentives to potential trial participants on recruitment rates (SWAT 59)?
6. What is the effect of mentioning scarcity of trial places in invitation letters on recruitment of trial participants (SWAT 60)?
7. What is the effectiveness of telephoning people who do not respond to a postal invitation on recruitment to randomised trials (SWAT 61)?

Retention strategies
1. What is the effect of adding a pen printed with the trial/university logo to the trial invitation on retention rates (SWAT 37)?
2. What is the effectiveness of a theoretically informed cover letter on improving response rates to annual postal questionnaires (SWAT 24)?
3. What is the effect of a text message notification versus no text message on questionnaire response rates (SWAT 25/SWAT 31)?
4. What is the effectiveness of a personalised text message versus a standard text message for promoting response to postal questionnaires (SWAT 35)?
5. What is the effect of timing text message prompts to increase trial participant response to postal questionnaires (SWAT 44)?
6. What is the effectiveness of sending pre-notification cards (letters/email) to trial participants 1-month (2 weeks) before outcome measurement to improve retention (SWAT 76/SWAT 86)?
7. What is the impact of receiving a social incentive strategy cover letter compared with a standard covering letter on response to postal questionnaires? (SWAT 144)
8. Do courtesy telephone calls to trial participants following enrolment increase future retention rates? (SWAT 114)

The PROMETHEUS programme built on the work of the UK MRC START programme through expansion of the number of different recruitment and retention strategies evaluated. For example, UK MRC START developed and evaluated only two recruitment strategies (a revised participant information leaflet and a multimedia strategy), which were evaluated in primary and community care trials only and resulted in many host RCTs that expressed an interest in doing one of the SWATs (57%, *n* = 37) being excluded due to their recruitment methods being unable to accommodate the implementation of a strategy or its evaluation. Therefore, PROMETHEUS prioritised a broad list of recruitment and retention strategies that could be evaluated, and host trials could also evaluate their own strategies should they wish.

For the PROMETHEUS programme, host trials were encouraged to evaluate one of the strategies listed in Table 1, or an adapted version and/or a novel recruitment/retention strategy, provided the strategy was evaluated using a randomised design. Host trial teams were able to embed multiple single SWATs or SWATs of a factorial design.

Studies Within a Trial number correlates to the registration number listed on the Northern Ireland Network for Trials Methodology Research SWAT repository

### Host trial support and funding

Eligible host trials were mainly identified and recruited through the collaborator CTUs and the programme’s advertisement on the University of York Trials Unit web page, through emails to all registered CTUs in the UK, and through conference presentations. These teams were invited to apply for funding of up to £5000 for each SWAT they embedded in their host trial.

To be eligible, host RCTs were also required to meet the following criteria:

(1). Registered or eligible for registration on the UK Clinical Research Network Portfolio
(2). In the planning phase; recruiting or following up participants, or be in the process of applying for ethics permission
(3). Willing to apply for ethics permission or amendment to undertake at least one SWAT of a recruitment or retention strategy
(4). Willing to randomise and deliver the recruitment or retention strategy according to a shared protocol and share data with the SWATs team and help to write up findings for publication
(5). Willing to use or register their SWAT on the UK MRC Northern Ireland Hub for Trial Methodology SWAT Repository, a free-to-use online database of ongoing SWATs if the strategy being evaluated is not already registered (SWAT store: www.qub.ac.uk/SWAT-SWAR)
(6). Able to provide evidence of funding for the host trial (such as a letter from the funder)
(7). Provide patient level data to the PROMETHEUS team to allow individual patient level meta-analysis

Three independent members of the PROMETHEUS programme, including both the programme members located at York Trials Unit and PROMETHEUS co-applicants, peer-reviewed each host trial application -and protocol to ensure methodologically robust replicable research was planned. Reviewers were asked to report their peer review comments and scores using a Peer Review Assessment Form (Appendix 1), which was adapted from the peer review form used by the HRB-TMRN to assess SWAT funding applications.^19^ Host trials were assessed on both their host trial and SWAT registrations, their willingness to attain suitable SWAT approvals, their agreement to share SWAT data to aid the publishing of SWAT results and their current study phase. For each funding application, the same peer reviewers were asked to review the proposed SWAT protocol within 2 weeks of the application being received, and to comment on the following: (1) Eligibility, (2) Priority and scientific quality, (3) Costings, and (4) Overall rating of the application, where a score of 1 indicated ‘Recommend to fund the SWAT’, 2 indicated ‘Recommend to fund the SWAT subject to changes and clarification’, and 3 indicated ‘Do not recommend funding’ (as detailed in Appendix 1).

The PROMETHEUS team supported host trials to identify appropriate recruitment and/or retention strategy/strategies. This was achieved through individual meetings with host trial teams to further discuss the host trial’s initial thoughts on strategies to include, and to then refine this further if required. Further support was also provided throughout the project including assistance in writing SWAT protocols; provision of templates and guidance in achieving Research Ethics Committee (REC) approval; guidance on writing and submitting SWATs for publication.

As the opportunity arose, we also tested the methodological feasibility of ‘simultaneous SWAT’ design, which involves the co-ordinated implementation of a specific SWAT strategy within multiple pre-identified host trials concurrently. For simultaneous SWATs, REC approval only needs to be obtained once to allow SWAT implementation within all of the included host trials and the results from each host trial are reported simultaneously within one publication, allowing a more rapid increase of the evidence base. An example of this is the Christmas card SWAT (SWAT 82; Table 2), for which only one ethics application was submitted for the SWAT to be implemented within eight host trials. These SWATs differ to identical SWATs which are instead conducted within separate host trials at the same time and each require an individual REC approval. The evaluation of personalised SMS reminders vs no reminder (SWAT 35; Table 2), is an example of an identical SWAT implemented within three separate host trials.

**Table 2.** Details of the retention SWATs funded by the PROMETHEUS programme.

Strategy	Number of host trials	SWAT protocol number^a^
Responsive SMS vs non-responsive SMS	1	SWAT 109
Personalised SMS vs non-personalised SMS	3	SWAT 35
Personalisation and timing of SMS factorial SWAT	2	SWAT 35 and SWAT 44
Personalised, early SMS vs personalised, late SMS vs non-personalised, early SMS vs non-personalised, late SMS		
Pre-notification cards vs no notification card	3	SWAT 76 and SWAT 86
Generic thank you card vs personalised thank you card vs no card	1	SWAT 54
Christmas card vs no Christmas card^b^	8	SWAT 82
Thank you phone call vs thank you post card	1	SWAT 121
Courtesy thank you phone call vs thank you post card vs no additional strategy	1	SWAT 114
Thank you pre-notification e-mail/letter vs no communication	1	SWAT 77
Pen included with study questionnaire vs no pen	2	SWAT 92
Pen and social incentive cover letter factorial SWAT	1	SWAT 92 and SWAT 144
Pen vs pen and social incentive cover letter (in addition to standard cover letter) vs social incentive cover letter vs standard cover letter only		
Primary outcome printed on pink paper vs printed on white paper	1	SWAT 110
Conditional financial incentives vs unconditional financial incentives	1	SWAT 96

^a^Northern Ireland SWAT repository store protocol registration number.

^b^undertaken as a simultaneous SWAT.

SMS: Short Message Service.

Of the 42 total SWATs, the PROMETHEUS programme successfully funded two simultaneous SWATs. These two specific SWAT strategies evaluated the effect of clinician recruitment training on participant recruitment and the sending of Christmas cards to participants on participant retention (SWAT 82 on the Northern Ireland SWAT repository), with them each being implemented within four and eight host trials, respectively^20^ (Table 3).

**Table 3.** Details of the recruitment SWATs funded by the PROMETHEUS programme.

Strategy	Number of host trials	SWAT protocol number^a^
Recruitment training for trial staff vs no recruitment training^b^	4	SWAT 111
Participant invitation letter with personal wet signature vs generic signature	1	Registration in progress
Participant study invitation including a generic doctor-patient photograph vs including no photograph	1	SWAT 53
Brief PIL vs standard length PIL	1	SWAT 137
PIL and a pictorial aid of the randomisation process vs PIL without a pictorial aid	2	SWAT 102
Optimised PIS vs conventional PIS	1	SWAT 101
Pen and PIL factorial SWAT Pen vs pen and a brief PIL vs a brief PIL vs no additional strategies	1	SWAT 137

^a^Northern Ireland SWAT repository store protocol registration number.

^b^Undertaken as a simultaneous SWAT.

PIL: Patient Information Leaflet.

## Results

**Figure 1.**
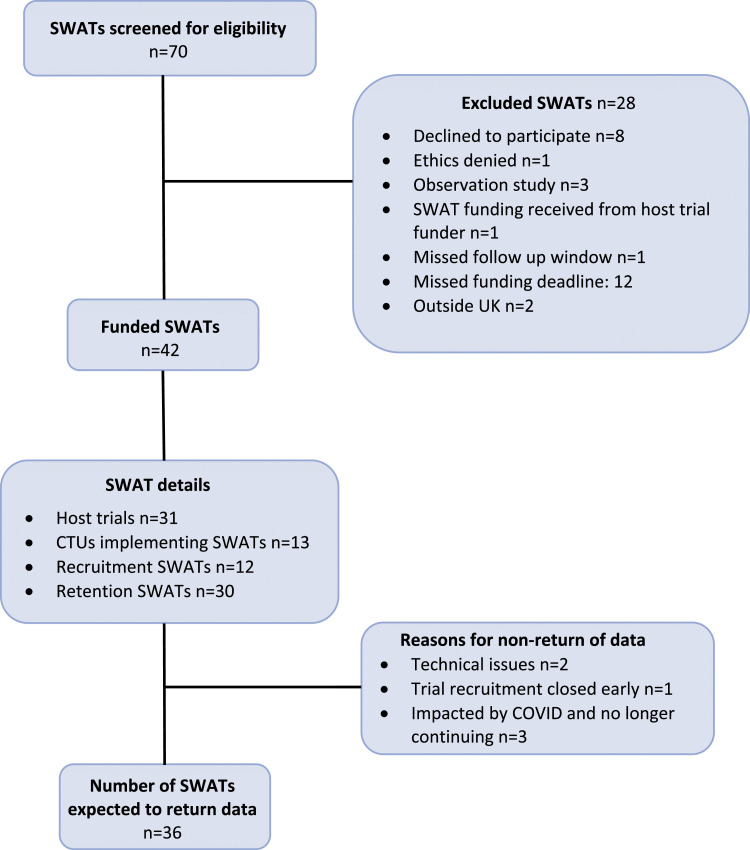
Flow chart of SWATs funded by the PROMETHEUS programme.

The PROMETHEUS programme commenced in April 2018 and was due to finish in September 2020. A no-cost extension of the programme was granted by the UK MRC to April 2021, to take into account delays occurring due to the COVID 19 pandemic. During this period, 42 SWATs were supported, where each contributing SWAT was counted separately for factorial and simultaneous designs (e.g. a 2 × 2 factorial SWAT is classed as two SWATS). These 42 SWATs were implemented within 31 host trials across 12 CTUs, as seen in Figure 1. Together these 31 host trials span 17 different health research areas (Table 4). Five of the host trials implemented more than one SWAT, and a further four each implemented a factorial design SWAT, allowing for an assessment of two SWATs simultaneously (see Appendix 2 for details of the host trials). In total, 12 of the funded SWATs evaluated recruitment strategies and 30 SWATs evaluated retention strategies. Results are expected for 36 SWATs as six SWATs could not be completed; two SWATs encountered technical issues, meaning that their SWAT intervention was not implemented correctly and therefore had to be abandoned, one SWAT was embedded in a host trial that terminated recruitment early due to having already answered its question and three further SWATs could not proceed due to their host trial being forced to change its method of following up participants as a result of the COVID-19 pandemic.

**Table 4.** The host trial research area of each of the SWATs funded by the PROMETHEUS programme.

Research area	Host trials	Number of host trials	Number of SWATs
Surgical	ACTIVE, DISC^a^, L1FE, ProFHER-2^a^, START:REACTS, UK FROST, C-Gall, MAGIC, PUrE	9	11
Fall reduction	OTIS^a^, SSHew	2	3
Orthopaedic (rehabilitation)	ARTISAN, KREBS^a^	2	3
Respiratory	CLEAR^a^	1	3
Smoking cessation	CPIT-III, MiQuit-3^a^	2	3
Wound care	SWHSI-2^a^	1	3
Oncology (screening and treatment)	IntAct, POSNOC^a^, ActWELL	3	3
Community pharmacy	CHAMP-1^a^	1	2
Physiotherapy	PEP-TALK, GRASP	2	2
Primary care (Signs and symptoms)	MSS3^a^	1	2
Rheumatology	TOPaZ, WORKWELL	2	2
Urology	FUTURE, SARC	2	2
Oral health	REFLECT	1	1
Gastrointestinal	IBD-BOOST	1	1
Gynaecology	VITA	1	1

^a^Host trials which implemented more than 1 SWAT.

The PROMETHEUS team classified the trials into ‘research area’ categories after referring to the category assigned to each trial on ISRCTN https://www.isrctn.com/

The cost of funding requested for a SWAT was £2,600, calculated from 29 SWATs, two of which were factorial SWATs, and each factorial SWAT was classed as one SWAT within this calculation (range £500 to £5000). The simultaneous SWATs (*n* = 12 separate SWATs) were not included as their cost does not representative the cost of a single SWAT and a further SWAT requested no funding after applying due to their requested funds being so low (approximately £300). This can be broken down into an average of £867 for consumables (data from 15 SWATs) and £1753 for staff time (data from 13 SWATs) – it was not always possible to distinguish staff and consumable costs from the funding applications. It is anticipated that both the overall cost and that linked to staff time may be an underestimate of the true cost of SWATs due to the large proportion of SWATs being done at York Trials Unit (YTU), where the PROMETHEUS programme was based, and as such costs for staff time were not included. When excluding YTU SWATs, the average cost rises to £3535 - with average staff cost being £2359 (data from 17 and 9 SWATs respectively). Both the simultaneous Christmas card SWAT (SWAT 82 on the Northern Ireland SWAT repository) and the Training SWAT^20^ were not included in the above calculations due to how they were performed, making them unrepresentative of the cost of a single SWAT. For instance, the method requires only one analysis and write up time, as opposed to the 8 and 4 respectively that would have been required if these SWATs were undertaken independently, thus increasing cost-efficiency.

At the time of writing, 22 of the total 42 PROMETHEUS funded SWATs have been completed, of which the results for seven SWATs has been published (Appendix 2). The results of the remaining 15 completed SWATs are currently in draft or under review. The results of all ongoing SWATs will be published following their completion. All publications from the PROMETHEUS programme will be detailed on the University of York, PROMETHEUS website.^21^ Publications of funded SWATs will also be shared with the lead authors of the Cochrane systematic reviews of recruitment and retention strategies.

## Discussion

### Key findings

Overall, the PROMETHEUS programme successfully embedded 42 SWATs within 31 host RCTs, exceeding the programme’s original target of embedding 25 SWATs. This was possible due to the majority of the SWATs costing less than the proposed funding limit of £5,000, with the average SWAT cost being £6465 less than the £10,000 SWAT funding made available by the Health Technology Assessment. Therefore, PROMETHEUS is currently the largest programme of work to act as a central coordination point to offer both funding and practically support the embedding of SWATs within trials, independently contributing the largest amount of evidence to the recruitment and retention strategy evidence base. These SWATs were collectively implemented within a large number of host trials across multiple clinical trials units, demonstrating the wide reach of the programme. The programme’s success confirms the feasibility of implementing methodological research within a vast range of research areas when appropriate resource and infrastructure support is made available. It also confirms the feasibility and acceptability among trial teams of conducting simultaneous SWATs, enabling a more rapid evaluation of recruitment and retention strategies.

### Strengths

As detailed in Tables 3 and 2, the PROMETHEUS programme has contributed substantially to the evidence base of many of the recruitment and retention strategies, as well as developing methodological innovations by establishing the feasibility of undertaking simultaneous SWATs of both a trial staff recruitment training strategy on participant recruitment and the sending of a Christmas card on participant retention across multiple host trials. Large contributions to the retention evidence base have also been made for both the inclusion of pens within participant study questionnaires and the use of text messages on participant retention;^22–26^ the findings for which have both been added to the most recent Cochrane retention review.^9^ Meta-analyses within this review show the inclusion of a pen compared with no pen and use of an electronic prompt compared with no prompt provide a risk difference of 2% (95% CI 0%–4%) and 2% (95% CI 1%–6%) respectively.

As host trial teams were often inexperienced in conducting and implementing SWATs, the PROMETHEUS programme acted as an invaluable coordination point, providing teams with the confidence and knowledge to do so. Further to this, the programme identified that the lack of SWAT funding was often a barrier to implementation; a concept which was reinforced as the number of conducted SWATs increased following the introduction of PROMETHEUS funding. Some host trial teams embedded more complex SWAT designs, such as the recruitment Training SWAT.^20^ However, in light of the PROMETHEUS programme providing host trial teams with both funding and guidance, the encouragement of external researchers to conduct a SWAT proved difficult; with the majority of the SWATs being conducted by both York Trials Unit (18 SWATs, 43%) and the CTUs which PROMETHEUS co-applicants were associated with (16 SWATs, 38%). We found a greater interest and conduct of SWATs evaluating retention, rather than recruitment strategies. This may be due to retention SWATs being potentially less challenging to undertake than recruitment SWATs, as logistically, there is likely to be more time to introduce a retention strategy when there are multiple follow-up time points. Alternatively, there may also be greater time pressure associated with the embedding of a recruitment rather than retention SWAT, due to the additional tasks of site set up. Lessons learned from the PROMETHEUS programme are also discussed by Clark et al.^27^

### Limitations

In response to the COVID-19 pandemic, the recruitment pathway of many of the host trials within which PROMETHEUS funded SWAT were embedded were either paused or altered; negatively impacting many of the SWATs. Unfortunately, this led to several SWATs being either delayed or prematurely terminated. Further to this, the funded SWATs evaluated a wide range of recruitment and/or retention strategies. While this led to an overall increase in the evidence base, the evidence generated did not conclude the effectiveness of all SWAT strategies evaluated.

In light of the programme exceeding its target in relation to the number of SWATs embedded within host trials, the majority of these SWATs were conducted by researchers who were already engaged, thus meaning that the programme had limited engagement with the wider research community. As all of the funded SWATs were also conducted within the UK, the SWAT evidence generated through PROMETHEUS is not necessarily applicable to populations within other countries.

### Comparison with earlier research

Building on and improving on the SWAT work initiated by UK MRC START, which established the feasibility of testing recruitment strategies across multiple host trials,^11,12^ PROMETHEUS has successfully undertaken more SWATs at a faster pace, answered a broader and more strategic range of questions around both recruitment and retention, and successfully disseminated findings. However, PROMETHEUS faced challenges that were similar in UK MRC START, such as a large proportion of the SWATs being undertaken by researchers linked with the PROMETHEUS team. There are a range of reasons for the success of PROMETHEUS, which includes prior learning on SWATs gained from UK MRC START and other work undertaken by its collaborators, the financial support to undertake SWATs, as well as the support mechanism provided to host trial teams from a well-established registered Clinical Trials Unit.

### Future research

Future research needs to focus on identifying gaps in the evidence base and targeting these to reduce the areas of uncertainty for the effectiveness of recruitment and retention strategies. For certain strategies this may require simultaneous, or coordinated SWATs, designed to rapidly provide definitive answers to questions across multiple host trials at the same time. Where possible these future evaluations should be performed simultaneously within multiple host trials, to allow for rapid evidence collection, as the PROMETHEUS programme has shown this approach to be feasible. Future work should consider issues around implementation of SWATs to enable the wider trials community to undertake, report and adopt the findings of SWATs. Chief investigators should be encouraged to consider the embedding of a SWAT at the funding stage. Further discussion in relation to the challenges and solutions for the embedding of SWATs is offered by Arundel et al.^28^

Further to this, it is important that SWAT specific funding streams are identified to continuation of work such as that reported here and the identification of both effective and ineffective recruitment and retention strategies. As the evidence base develops, it will become increasingly important for trialists to utilise the evidence base in a systematic way to identify both effective and ineffective strategies. Further work surrounding dissemination and implementation of SWAT evidence will also be required.

Finally, as higher certainty evidence starts to come from SWAT evaluations and meta-analysis of results of SWATs, attention needs to be given to ensuring that this evidence is used by trialists. Simple publication of SWAT results is unlikely to be sufficient to change behaviour and more effective dissemination strategies and incentives will need to be designed and implemented.

## Conclusion

When SWAT funding was made available, we found that many teams embedded SWATs into their research. Having a central point of contact that coordinated SWAT activity alongside providing funding has been key in determining the success of PROMETHEUS. Simultaneous SWATs can be successfully embedded, and we recommend these are undertaken in the future to increase the evidence rapidly.

## Supplemental Material

Supplemental Material - PROMoting the use of studies within a trial (PROMETHEUS): Results and experiences from a large programme to evaluate the routine embedding of recruitment and retention strategies within randomised controlled trials routinely Supplemental Material for PROMoting the use of studies within a trial (PROMETHEUS): Results and experiences from a large programme to evaluate the routine embedding of recruitment and retention strategies within randomised controlled trials routinely by Laura Doherty, Adwoa Parker, Catherine Arundel, Laura Clark, Elizabeth Coleman, Catherine Hewitt, David Beard, Peter Bower, Paul Brocklehurst, Cindy Cooper, Lucy Culliford, Declan Devane, Richard Emsley, Sandra Eldridge, Sandra Galvin, Katie Gillies, Alan Montgomery, Chris Sutton, Shaun Treweek, David Torgerson in Research Methods in Medicine & Health Sciences
