# Supplementary material for: PROMoting the use of studies within a trial (PROMETHEUS): Results and experiences from a large programme to evaluate the routine embedding of recruitment and retention strategies within randomised controlled trials routinely
Source: Res Methods Med Health Sci. 2022 Dec 22;4(3):113–22. doi: 10.1177/26320843221147841 (PMC13020956; doi:10.1177/26320843221147841)
Supplement: Supplemental Material - PROMoting the use of studies within a trial (PROMETHEUS): Results and experiences from a large programme to evaluate the routine embedding of recruitment and retention strategies within randomised controlled trials routinely [file sj-pdf-1-rmm-10.1177_26320843221147841.pdf]

Appendices

Appendix 1

Promoting the use of SWATs (PROMETHEUS): Peer review assessment form

Reviewer name: ______________________________

Host trial name: ______________________________

Rating of applications received

- 1 = recommend funding
- 2 = recommend funding subject to changes and clarifications
- 3 = do not recommend funding

Reviewer's rating: _______

Reviewers' comments (Maximum 250 words):

Reviewer's signature: ____

DATE: __ / __ / _____

Guidance notes for reviewers

Please consider the following when undertaking the peer review:

1. **Eligibility**

To be eligible, host trials will be:

- Registered or eligible for registration on the UK Clinical Research Network Portfolio.
- In the planning phase, be in the process of applying for ethics permission, or recruiting or following up participants.
- Willing to apply for ethics permission or amendment to undertake at least one SWAT of a recruitment or retention intervention.
- Willing to randomise and deliver the recruitment or retention intervention according to a shared protocol and share data with the MRC SWATS team and help to write up findings for publication.
- Willing to use or register their SWAT on the MRC-HTMR All-Ireland Hub website, if the intervention being evaluated is not already registered.

2. Priority and scientific quality:

- Appropriateness of the research design.
- Appropriateness of the research methods.
- Feasibility of the proposed SWAT (including recruitment and retention of participants, project timeline, etc.).
- Does/do the proposed interventions(s) match our current list of key questions (below). If SWAT intervention(s) are not currently on the list, or do you deem the SWAT will make a useful contribution to the evidence base?

Table 1: List of key recruitment and retention questions

**Recruitment interventions**
1. What is the effect of adding a pen printed with the trial/university logo to the trial invitation on recruitment rates (SWAT 37)?
2. What is the impact of recruitment sites receiving an extra trial co-ordinator visit on recruitment rates? (SWAT 27)
3. What is the effectiveness of a brief participant information leaflet (PIL) versus standard length PIL on participant recruitment rates?
4. What is the impact of a training workshop for staff recruiting patients into trials on recruitment rates?
5. What is the effect of offering financial incentives to potential trial participants on recruitment rates? (SWAT 59)
6. What is the effect of mentioning scarcity of trial places in invitation letters on recruitment of trial participants? (SWAT 60)
7. What is the effectiveness of telephoning people who do not respond to a postal invitation on recruitment to randomised trials? (SWAT 61)
**Retention Interventions**
1. What is the effect of adding a pen printed with the trial/university logo to the trial invitation on retention rates (SWAT 37)?
2. What is the effectiveness of a theoretically informed cover letter on improving response rates to annual postal questionnaires? (SWAT 24)

3. What is the effect of a text message notification versus no text message on questionnaire response rates? (SWAT 25/SWAT 31)
4. What is the effectiveness of a personalised text message versus a standard text message for promoting response to postal follow-up questionnaires? (SWAT 35)
5. What is the effect of timing text message prompts to increase trial participant response to postal questionnaires? (SWAT 44)
6. What is the effectiveness of sending pre-notification cards to trial participants 1-month before outcome measurement to improve retention.
7. What is the impact of receiving a social incentive intervention cover letter compared with a standard covering letter on response to postal questionnaires? (SWAT registration submitted)
8. Do courtesy telephone calls to trial participants following enrolment increase future retention rates?

3. Costing

- Is the funding requested appropriate for the type of SWAT proposed? (e.g. staff time, intervention costs such as printing or pens, conference costs).
- We cannot pay for open access fees as these are paid as a block grant to Higher Education Institutions.

4. Rating of applications received

- 1 = recommend funding
- 2 = recommend funding subject to changes and clarifications
- 3 = do not recommend funding

Appendix 2

Host trial title	Host trial acronym	Host trial protocol	SWAT	Published SWAT data
External frame versus internal locking plate for articular pilon fracture fixation: a multi-centre randomised controlled trial	ACTIVE	https://www.journalslibrary.nihr.ac.uk/programmes/hta/1513084#/	SMS vs responsive SMS (retention)	
A randomised control trial to assess the impact of a lifestyle strategy (ActWELL) in women invited to NHS breast screening	ActWELL	https://bmjopen.bmj.com/content/8/11/e024136	Pre-notification cards vs no notification card (retention)	https://doi.org/10.12688/f1000research.50890.2
Acute Rehabilitation following traumatic anterior shoulder dislocation	ARTISAN	https://bmjopen.bmj.com/content/10/11/e040623	Thank you phone-call vs thank you post card (retention)	
A randomised controlled trial comparing laparoscopic cholecystectomy with observation/conservative management for preventing recurrent symptoms and complications in adults with uncomplicated symptomatic gallstones	C-GALL	https://research-information.bris.ac.uk/en/publications/protocol-for-a-randomised-controlled-trial-comparing-laparoscopic	Christmas card vs no Christmas card (retention)	https://doi.org/10.1136/bmj-2021-067742
A multi-centre, cluster randomised controlled, open pilot trial to establish the feasibility of conducting a large-scale study comparing an strategy discussing alcohol within routine medication consultations with usual care in community pharmacies.	CHAMP-1	https://doi.org/10.1186/s12913-020-05797-z	Factorial design SWAT (retention):<br><br>Personalised, early SMS vs personalised, late SMS vs non-personalised, early SMS vs non-personalised, late SMS	
A 2x2 factorial randomised open label trial to determine the clinical and cost-effectiveness of hypertonic saline (HTS 6%) and carbocisteine for airway clearance versus usual care over 52 weeks in bronchiectasis	CLEAR	https://doi.org/10.1186/s13063-019-3766-9	Three separate SWATs:<br><br>Participant invitation letter with personal wet signature vs generic signature (recruitment).<br><br>Participant study invitation including a generic doctor-patient photograph vs including no photograph (recruitment).	https://doi.org/10.12688/f1000research.75339.1

			Generic thank you card vs personalised thank you card vs no card (retention).	
The smoking cessation in pregnancy incentives trial	CPIT-III	https://doi.org/10.1186/s13063-019-4042-8	Christmas card vs no Christmas card (retention).	https://doi.org/10.1136/bmj-2021-067742
Dupuytren's strategys surgery vs collagenase	DISC	https://doi.org/10.1186/s13063-021-05595-w	Two separate SWATs:<br><br>Christmas card vs no Christmas card (retention).<br><br>Recruitment training for trial staff vs no recruitment training (recruitment).	https://doi.org/10.1136/bmj-2021-067742<br><br>https://doi.org/10.1177/26320843221106950
Female Urgency, Trial of Urodynamics as Routine Evaluation; a superiority randomised clinical trial to evaluate the effectiveness and cost effectiveness of invasive urodynamic investigations in management of women with refractory overactive bladder (OAB) symptoms	FUTURE	https://www.fundingawards.nihr.ac.uk/award/15/150/05	Christmas card vs no Christmas card (retention).	https://doi.org/10.1136/bmj-2021-067742
Getting it Right: Addressing Shoulder Pain	GRASP	http://dx.doi.org/10.1136/bmjopen-2017-018004	Personalised SMS vs non-personalised SMS (retention).	https://doi.org/10.1186/s13063-021-05452-w
Living well with inflammatory bowel disease: optimising management of symptoms of fatigue, abdominal pain, and faecal urgency/incontinence via tailored online self-management	IBD-BOOST	10.21203/rs.3.rs-62886/v1	Brief PIL vs standard length PIL (recruitment).	
Intraoperative Fluorescence Angiography to Prevent Anastomotic Leak in Rectal Cancer Surgery	IntAct	https://www.virtualpathology.leeds.ac.uk/clinical/colorectal/intact/docs/IntAct_protocol_v2.0_20170718.pdf	Recruitment training for trial staff vs no recruitment training (recruitment).	https://doi.org/10.1177/26320843221106950
A Randomised Controlled Trial of the effect of a Two-layer Compression Bandage System on Knee Function following Total Knee Arthroplasty	KReBs	https://doi.org/10.1186/s13063-019-3344-1	Two separate SWATs:<br><br>Personalised SMS vs non-personalised SMS (retention).<br><br>Pen included with study questionnaire vs no	https://doi.org/10.12688/f1000research.23018.1<br><br>https://doi.org/10.12688/f1000research.24244.1

			pen (retention).	
Lateral Compression Type-1 Fracture Fixation in the Elderly, a Randomised Controlled Trial	L1FE		Courtesy thank you phone-call vs thank you post card vs no additional strategy (retention).	
Melatonin for Anxiety prior to General anaesthesia In Children	MAGIC	https://fundingawards.nihr.ac.uk/award/16/80/08	Personalised SMS vs non-personalised SMS (retention).	
RCT and meta-analysis testing effectiveness and cost-effectiveness of a tailored text message programme for smoking cessation in pregnancy	MiQuit-3	https://doi.org/10.1186/s13063-019-3341-4	Factorial design (retention):<br><br>Personalised, early SMS vs personalised, late SMS vs non-personalised, early SMS vs non-personalised, late SMS	https://doi.org/10.12688/f1000research.51964.1
Multiple Symptoms Study 3: pragmatic trial of a community-based clinic for patients with persistent (medically unexplained) physical symptoms	MSS3	https://fundingawards.nihr.ac.uk/award/15/136/07	Factorial design (recruitment):<br><br>Pen vs pen and a brief PIL vs a brief PIL vs no additional strategys	
Does Occupational Therapist led environmental assessment and modification reduce falls among high-risk older people	OTIS	10.1136/bmjopen-2018-022488	Factorial design (retention):<br><br>Pen vs pen and social incentive cover letter (in addition to standard cover letter) vs social incentive cover letter vs standard cover letter only	https://doi.org/10.12688/f1000research.23767.1
A behaviour change physiotherapy strategy to increase physical activity following hip and knee replacement: a pragmatic phase II randomised controlled trial	PEP-TALK	http://dx.doi.org/10.1136/bmjopen-2019-035014	Primary outcome printed on pink paper vs printed on white paper	https://doi.org/10.1177/26320843221074344
POsitive Sentinel NOde: adjuvant therapy alone versus adjuvant therapy plus Clearance or axillary radiotherapy	POSNOC	http://www.posnoc.co.uk/healthcare-professionals/protocol.aspx	PIL with a pictorial aid to show the randomisation process vs PIL without a pictorial aid (recruitment)	
Proximal Fracture of the Humerus: Evaluation by Randomisation - 2	ProFHER-2	10.1186/1471-2474-10-140	Two separate SWATs:<br><br>Christmas card vs no Christmas card (retention).<br><br>Recruitment training for trial staff vs no recruitment training (recruitment).	 <br>https://doi.org/10.1136/bmj-2021-067742<br><br>https://doi.org/10.1177/26320843221106950

The clinical and cost effectiveness of surgical strategys for stones in the lower pole of the kidney: The Percutaneous nephrolithotomy, flexible Ureterorenoscopy and Extracorporeal shockwave lithotripsy for lower pole kidney stones Randomised Controlled Trial (PUrE RCT)	PUrE-RCT	https://w3.abdn.ac.uk/hsru/pure/Public/Download.aspx?ID=4	Christmas card vs no Christmas card (retention).	https://doi.org/10.1136/bmj-2021-067742
A Randomised controlled trial to Evaluate the effectiveness and cost benefit of prescribing high dose FLuoride toothpaste in preventing and treating dEntal Caries in high-risk older adulTs.	REFLECT	10.1186/s12903-019-0749-x	Christmas card vs no Christmas card (retention).	https://doi.org/10.1136/bmj-2021-067742
Salbutamol for analgesia in renal colic: A prospective, randomised, placebo-controlled Phase II trial (SARC)	SARC		Optimised PIS vs conventional PIS (recruitment)	
The SSHeW study - Stopping slips among healthcare workers: A research study about slip resistant footwear in the NHS workplace	SSHew	http://dx.doi.org/10.1136/bmjopen-2018-026023	Pen included with study questionnaire vs no pen (retention)	https://doi.org/10.12688/f1000research.23651.1
START:REACTS: Sub-acromial spacer for Tears Affecting Rotator cuff Tendons: a Randomised, Efficient, Adaptive Clinical Trial in Surgery.	START:REACTS	http://dx.doi.org/10.1136/bmjopen-2020-036829	Recruitment training for trial staff vs no recruitment training (recruitment).	https://doi.org/10.1177/26320843221106950
A pragmatic multicentre randomised controlled trial to assess the clinical and cost effectiveness of negative pressure wound therapy versus usual care for surgical wounds healing by secondary intention	SWHSI-2		Three separate SWATs:<br><br>PIL and a pictorial aid of the randomisation process vs PIL without a pictorial aid (recruitment)<br><br>Thank you card vs no thank you card (retention)<br><br>Christmas card vs no Christmas card (retention).	https://doi.org/10.1136/bmj-2021-067742
Treatment of Osteogenesis Imperfecta with Parathyroid hormone and Zoledronic acid	TOPaZ	https://clinicaltrials.gov/ct2/show/NCT03735537	Pre-notification cards vs no notification card (retention)	

United Kingdom Frozen shoulder trial	UK FROST		Pre-notification card vs post-notification card (retention)	https://doi.org/10.1016/j.jclinepi.2020.03.001
Metronidazole versus lactic acid for treating bacterial vaginosis	VITA	https://trialsjournal.biomedcentral.com/track/pdf/10.1186/s13063-019-3731-7.pdf	Conditional financial incentives vs unconditional financial incentives (retention)	
A Randomised Controlled Trial of Job Retention Vocational Rehabilitation for Employed People with Inflammatory Arthritis: the WORKWELL Trial.	WORKWELL	https://doi.org/10.1186/s12891-020-03619-1	Thank you pre-notification e-mail/letter vs no communication (retention)	https://doi.org/10.1177/26320843221098427
